# A Multispecialty Evaluation of Thiel Cadavers for Surgical Training

**DOI:** 10.1007/s00268-016-3868-4

**Published:** 2017-01-31

**Authors:** Marina Yiasemidou, David Roberts, Daniel Glassman, James Tomlinson, Shekhar Biyani, Danilo Miskovic

**Affiliations:** 1 The John Goligher Academic Surgical Unit, St. James University Hospital, Level 7, Clinical Science Building, Beckett Str., Leeds, YSW LS9 7TF UK; 2 Department of Urology, St James University Hospital, Beckett Street, Leeds, LS9 7TF UK; 3 School of Surgery, Health Education, Yorkshire and the Humber, Willow Terrace, University of Leeds, Leeds, LS2 9JT UK; 4 Division of Anatomy, Leeds Institute of Medical Education, School of Medicine, University of Leeds, Leeds, LS2 9JT UK

## Abstract

**Background:**

Changes in UK legislation allow for surgical procedures to be performed on cadavers. The aim of this study was to assess Thiel cadavers as high-fidelity simulators and to examine their suitability for surgical training.

**Methods:**

Surgeons from various specialties were invited to attend a 1 day dissection workshop using Thiel cadavers. The surgeons completed a baseline questionnaire on cadaveric simulation. At the end of the workshop, they completed a similar questionnaire based on their experience with Thiel cadavers. Comparing the answers in the pre- and post-workshop questionnaires assessed whether using Thiel cadavers had changed the surgeons’ opinions of cadaveric simulation.

**Results:**

According to the 27 participants, simulation is important for surgical training and a full-procedure model is beneficial for all levels of training. Currently, there is dissatisfaction with existing models and a need for high-fidelity alternatives. After the workshop, surgeons concluded that Thiel cadavers are suitable for surgical simulation (*p* = 0.015). Thiel were found to be realistic (*p* < 0.001) to have reduced odour (*p* = 0.002) and be more cost-effective (*p* = 0.003). Ethical constraints were considered to be small.

**Conclusion:**

Thiel cadavers are suitable for training in most surgical specialties.

## Introduction

Simulation has been a part of healthcare education for the past 50 years [1]. A significant amount of research demonstrates its value in surgical training [2, 3] and health authorities have identified simulation as a medium to improve patient safety [1, 4].

Aviation was one of the first industries to use simulation [5]. The philosophy was to create an environment as similar to the real one as possible. The pilots find themselves sitting within a cockpit, experiencing the same forces and audio-visual stimuli as in a real plane [6]. Conversely, the recreation of human organs that look and feel realistic still proves to be highly challenging [7].

Unlike the legislation they replaced, the Human Tissue Act 2004 and Human Tissue Act 2006 allow for surgical procedures to be performed on human cadavers in Britain [8]. This development opens up new horizons for high-fidelity surgical simulation. However, in a recent review, cadaveric surgical simulation was criticised, due to alteration of tissue quality caused by the embalming/preservation technique [9]. Simulation of advanced surgical operations in traditionally embalmed cadavers is often impossible, due to the rigidity of the tissue. Using fresh frozen cadavers is a popular option for such training. Another, less widespread alternative involves the use of soft-fix embalmed cadavers, such as those embalmed by the Thiel method, which preserves the live tissue texture and colour [10]. Thiel embalming fluid contains formaldehyde in a very low concentration, along with glycol, water and various salts, and hence is safer than a traditional embalming medium [11]. Following the embalming process, the tissue is preserved without the need for refrigeration or special storage facilities. Previous reports have highlighted the potential advantages of this technique for certain surgical specialties [12–15].

The aim of this study was to assess the suitability of Thiel cadavers for surgical training in a broad variety of surgical specialties. Previous studies have focused on a single type of surgery or technique; however, coordination between specialties would be needed to fully exploit the reusability advantage of Thiel cadavers [11]. Therefore, a cross-specialty consensus is essential.

## Materials and methods

The surgical academic unit of St. James’s University Hospital (SJUH) in collaboration with the Division of Anatomy and Mechanical Engineering, University of Leeds (UoL), organised a surgical dissection workshop, inviting consultant surgeons and senior trainees of all surgical specialties, to perform a wide variety of procedures on Thiel cadavers. The purpose of the workshop was to familiarise the participating surgeons with Thiel cadavers and capture their opinion on their use as high-fidelity “simulators” for surgical courses.

### Workshop structure

Both open and laparoscopic surgical instruments and equipment were available, as well as a microscope for spinal and neurosurgery. The cadavers were placed on theatre tables to facilitate accurate positioning.

The day started with introductory lecture delivered by an anatomist aiming to inform the audience about Thiel embalming. Immediately prior to the commencement of the first lecture, participants were presented with a questionnaire aiming to establish a “snapshot” opinion on the role of cadaveric dissection in surgical training prior to having dissection experience with Thiel cadavers.

The introductory lecture was followed by two dissection sessions and focus groups discussions. During the dissection, sessions surgeons were allowed to perform any surgical procedure they wished, and there were no structured activities during this part of the workshop. Shortly, after presenting the focus group results, participants were asked to complete a second questionnaire, basing their answers on their experience with Thiel cadavers during the workshop.

### Questionnaire

A consultant surgeon, expert in surgical education, (DM) and the surgical education fellow developed the questionnaire after reviewing relevant literature independently.

An initial meeting took place to identify the goals of the questionnaire. Following this, the two collaborators worked independently on questions to fulfil the set targets. Final questions were decided at a second consensus meeting and validated within a small group of trainees and consultants.

### Focus groups

Three focus groups were formed. The members of each group were decided in advance, maintaining constancy of specialties and expertise. The groups were presented with the same three questions: (1) what would motivate surgeons to use Thiel cadavers for surgical training (2) what would deter them and (3) what are the potential uses of Thiel cadavers in surgical training. Free discussion was assisted and coordinated by a facilitator. The results of the focus group discussions were validated by the entirety of the participants at a later stage.

### Cadavers

Three Thiel-embalmed cadavers were used for this study. The cadavers were donated for medical education and scientific research through the body donation programme of the UoL. The Thiel embalming protocol was based on that of Eisma et al. [16] which includes an initial perfusion stage followed by immersion in fluid for 2 months. The staff time for preparing Thiel cadavers is similar to that for preparing formalin-embalmed cadavers. Unlike, fresh frozen Thiel and formalin cadavers can be used in a considerably longer length in time (i.e. 3 years, at which time cadavers should be cremated) [16]. Therefore, preparation time is not expected to be a rate-limiting step for the use of Thiel cadavers in surgical training.

### Ethical approval

Institutional Review Board review was not needed, as the study did not involve living individuals. The study complied with the UK Human Tissue Act [8], the UK Human Tissue Authority Code of Practice on Anatomical Examination [17] and the Leeds Teaching Hospitals Trust/UoL Joint Policy for the Storage and Use of Human Tissue [18].

### Data collection and statistical analysis

Modified Likert scales (1: Not important at all–7: Very important, and 1: Completely disagree–7: Completely agree) were used for the questionnaire as appropriate. The Wilcoxon signed-rank test was used for comparing results of the pre- and post-workshop questionnaire. SPSS^®^ 17 was used for all statistical analyses. Levels of agreement were calculated using the intraclass correlation coefficient.

## Results

Of the forty invited, eighteen consultant surgeons and nine senior surgical trainees, from 10 different surgical specialties, and a consultant gastroenterologist (67.5%) (*n* = 27, all male) attended the workshop, representing nine surgical subspecialties. The median length of clinical experience in the according speciality was 6 years (11 for experts, 3 for trainees). The majority of participants had experience with surgical simulation both as faculty and students. All but two had experience with cadaveric simulation either as faculty or trainees during surgical simulation educational courses, but only a minority had worked with Thiel cadavers (*n* = 2) (Table 1).

**Table 1:** Participants’ experience with cadaveric dissection for surgical training

Gender	M:F 27:0					
Years of experience: median (range)	Consultants (surgeons who have completed their training): 11 (17)					
	Trainees: 3 (0)					
Experience using cadavers for surgical training as a trainer (faculty)—number of events participated in	No of events	None	1–2	3–5	6–10	>10
	No of surgeons	8	8	3	4	4
Experience using cadavers for surgical training as a trainee—number of event participated in	No of events	None	1–2	3–5	6–10	>10
	No of surgeons	8	6	3	5	5
What types of cadavers have you used before	Types of cadavers	None	Fresh/fresh frozen	Thiel	Formalin fixed	Others
	No of surgeons*	2	11	3	17	2

* Some surgeons had experiences with more than one types of cadavers. Range refers to maximum value minus minimum value

There was a high-level agreement among the participants that simulation is an important feature in surgical training and that a full-procedure simulator is important for all levels of surgical training as well as for senior surgeons learning new procedures (median score = 7, intraclass coefficient (ICC) = 0.765). The need for high-fidelity simulators was represented by high scores for questions on the realism of an ideal simulator, especially the accuracy of the anatomy (median = 6 (realistic feel), 6 (look), 5 (blood perfusion), 7 (gross anatomy) ICC = 0.921). Nevertheless, the satisfaction level with currently used simulation models was low, with a median score of 3.5 (65.4% were dissatisfied or undecided) (Table 2). The participants were given the option to elaborate on a free text box; however, no insight was given for which specific models the participants were satisfied or dissatisfied with. This question was aiming to evaluate whether a new training model should become available in the region.

**Table 2:** Surgeons’ opinion on simulation

Simulation related questions	Median score (range)	ICC
Satisfaction with existing simulators for their specialty	3.5 (6)	0.765*
Do you think simulation is important for surgical training?	7 (3)	0.765*
How important is a full-procedure simulation model for core surgical trainees?	6 (3)	0.765*
How important is a full-procedure simulation model for higher surgical trainees?	7 (5)	0.765*
How important is a full-procedure simulation model for consultants learning new procedures?	6 (4)	0.765*
*For a simulation model*		
How important is a realistic feel of tissues?	6 (5)	0.921**
How important is a realistic look of tissues?	6 (5)	0.921**
How important is active blood perfusion?	5 (5)	0.921**
How important is realistic anatomy of structures?	7 (2)	0.921**

A modified Likert scale was used for data collection (for first question: 1: completely disagree, 7: completely agree, for all other questions: 1: not important at all, 7: very important)

* Group 1 of questions for which ICC was calculated; ** Group 2 of questions for which ICC was calculated. Range refers to maximum value minus minimum value

### Specialty evaluation of Thiel cadavers

Answers to questions in the pre- and post-workshop questionnaires about the fidelity of Thiel cadavers as a simulation model were compared (Table 3). There was a statistically significant increase in scores regarding questions on the realism of the cadaveric tissue compared to live tissue after the workshop was completed (for both visual and haptic realism *p* < 0.001). After working on Thiel cadavers, odour was considered less of an issue than initially assumed (median pre = 5, post = 3, *p* = 0.002) and participants were increasingly convinced that the model is cost-effective (median pre = 5, post = 3, *p* = 0.003). The participants’ attitude towards the use of cadavers for surgical simulation was favourable (median pre = 2, post = 2, *p* = 0.015). The same was true for the feasibility of identifying significant surgical anatomical landmarks (median pre = 3, post = 2, *p* = 0.013). Ethical constraints in using cadavers for surgical education were considered to be small both before and after the workshop (median pre = 1.5, post = 1, *p* = 0.234). All Thiel-embalmed tissues (Table 4) and gross anatomy were considered to be realistic with the exception of the brain and the eyes.

**Table 3:** Opinion on cadaveric dissection before and after the Thiel open workshop

	Opinion before median (range)	Opinion after median (range)	*p* value
Cadaveric tissue feels significantly different from live tissue	6 (5)	3 (5)	<0.001
Cadaveric tissue looks significantly different from live tissue	6 (5)	3 (5)	<0.001
The use of cadavers for surgical simulation is of little benefit	2 (6)	2 (3)	0.015
It is difficult to identify anatomical landmarks in cadavers	3 (6)	2 (4)	0.013
Using cadavers for surgical training is unethical	1.5 (5)	1 (3)	0.234
Cadaveric dissection is expensive	5 (5)	4 (6)	0.003
The smell of cadavers can be disturbing	5 (6)	3 (6)	0.002

The answers were given on 1–7 Likert scale (1: completely disagree–7: completely agree)

**Table 4:** Non-specific realism of Thiel cadavers

Question	Median	Range
Tissue feels realistic	6	3
Tissue looks realistic	6	3
Gross anatomy is realistic	6	2
Skin	6	2
Fat	6	4
Muscle	6	3
Bone	6	3
Collagenous tissue (tendons)	6	3
Connective tissue planes (fascia)	6	2
Cartilage	6	3
Vessels	5	3

The answers were given on 1–7 Likert scale (1: not realistic at all–7: very realistic, 1, completely disagree, 7, completely agree)

### Focus group discussions

The main advantages of the Thiel cadavers identified were high level of visual and tactile realism, easy maintenance, versatility as a training tool and acceptable odour. The main disadvantage was the lack of blood flow and the poor realism of brain and eye tissues. The experts identified a number of potential applications in surgical training for each speciality. The surgeons focused on two areas: procedures for which no simulation model was available and procedures for which existing models are considered insufficient. For example, orthopaedic surgeons were impressed with the quality of the bone and suggested procedures that would make good use of that (e.g. joint replacements). Urologists suggested pyeloplasty and ureteric implantation. Currently, there is no available simulation model for the specific procedures. The focus groups also considered using Thiel cadavers for multidisciplinary teaching such as trauma courses. Finally, it was suggested that Thiel cadavers can be used to practice novel surgical approaches.

## Discussion

This study is the first to evaluate expert opinion on the use of Thiel cadavers across a broad range of specialities. Anatomical accuracy and fidelity of tissue properties were rated highly. Organ and tissue realism were excellent with three exceptions (i.e. brain, eyes and blood vessels). The results suggest that Thiel cadavers may be suitable for use in a broad range of surgical technical skills and human factors training. Issues traditionally associated with cadaveric simulation, such as disturbing odour, ethical constraints and cost, are believed to be less troubling using Thiel cadavers.

Animal models may be considered an alternative to cadaveric simulation due to the tissue texture and appearance may be similar to the one of the human body. However, the anatomy whilst similar is not identical [19] and UK law dictates that procedures can be performed on anaesthetised animals by appropriately trained individuals who bear a personal licence, hence creating a need for resources and time [20]. This study does not suggest that sole Thiel cadavers should be used for all surgical skills. Further research is required to match ideal types of simulation models to basic, moderate and advance surgical skills. This is beyond the scope of the current study.

The outcomes of this study are in line with previous comparative studies, which found Thiel cadavers ideally suited for teaching several surgical procedures [11, 12, 14, 15].

Embalming offers several advantages over the use of fresh frozen materials, including prevention of disease transmission and an ability to use the cadaver on multiple occasions and over a much greater length of time [11]. When comparing traditional formaldehyde fixation to Thiel embalming, the latter is considered a more realistic teaching model. The methodology used in these comparative studies was similar to that employed here.

Further, several non-comparative studies examined the feasibility and realism of Thiel-based simulation sessions. All concluded that Thiel cadavers provide an appropriate high-fidelity training platform for teaching the respective procedures [12, 14, 15, 21, 22]. Suitability of Thiel cadavers was established using either photographic evidence [12, 15] or a questionnaire [14].

As identified by the participating experts, Thiel bodies are also ideal for testing and fine tuning novel surgical approaches. Several studies used Thiel cadavers to prove feasibility of innovative surgical techniques. Heneweer et al. [23] described how they performed a laser-based minimally invasive procedure in order to recanalize chronically occluded femoral arteries, whilst Tokairin et al. [24] assessed the feasibility of mediastinoscopic subaortic and tracheobronchial lymph node dissection, utilising a new access route.

Both questionnaire and focus group discussion results indicate several advantages of Thiel over traditionally formalin-embalmed cadavers. These have been documented in studies conducted previously. Low odour is attributed to reduced formaldehyde concentration within the Thiel solution [10, 11], whilst life-like texture of tissue has been established during simulated operations [12–15, 25]. According to the experience of our anatomy institute, the immediate overall economic cost associated with a Thiel cadaver is 10–20% more than that of a formalin-fixed one. However, due to their “reusability” it is believed that in the long term Thiel is potentially more cost-effective.

Equally high-fidelity texture can be observed with fresh frozen cadavers; however, they have a very high initial and running cost, tissue quality deteriorates hourly and their dissection bears a risk of infection. Tissue deterioration with Thiel is significantly slower enabling their repeated usage, whilst their low storage demands may reduce running costs [26]. Finally, Thiel embalming prevents infection transmission [10].

Despite establishing good tissue fidelity for the majority of the cadaveric organs, eye and brain tissues were not found to be sufficiently realistic. As indicated by the focus group discussions, the experts were sceptical about the altered colour of the neurological tissue after Thiel embalming which could partially account for the reduced realism of the brain tissue. Nevertheless, feasibility studies of novel neurosurgical approaches have been successfully conducted using Thiel cadavers without concerns about the fidelity of neurological tissue [27, 28]. The exposure to room conditions [29] which could have led to decreased tissue plasticity, especially in the eyes could be a potential explanation. In a subsequent ophthalmology courses at our institution, eyes were injected with a gel-like material and the feedback received about the realism of the tissue properties was excellent. In addition, in the free text section, experts identified collapsed vessels and absence of bleeding, as well as lack of cerebrospinal fluid, as shortcomings. Whilst to our knowledge there is currently no solution for the latter, blood perfusion has been adequately simulated [30], though such an addition was not attempted during this workshop. Nevertheless, this technique for perfusion replication has some drawbacks such as time commitment (2 h), necessity for technical expertise, cost and inability to assess venous flow [30]. It is possible that injection of gel-like fluid could be beneficial in preventing the collapse of blood vessels but this has not been attempted in our laboratory.

The current study has some limitations. It is a snapshot validation study based on expert opinion, hence, does not provide objective data on tissue properties. However, expert and training surgeons are the ultimate potential end-users and it is paramount to gauge their opinion on a surgical simulation prototype before further exploring this modality. Furthermore, improvement of surgical skills and procedure-related knowledge after cadaveric dissection training course has been previously established in a series of studies [31–33]. A second limitation could be the possible introduction of bias through the introductory lecture. If any, this will be minimal as the lecture had a strictly informative nature and included no favouritism towards the Thiel embalming technique was expressed during it. The same approach was followed during the focus groups, during which no favouritism towards soft-embalmed cadavers was expressed and participants were asked to consider both advantages and disadvantages of cadaveric simulation. Finally, it should be noted that a number of potential participants invited to the session did not attend. These may have included surgeons with adverse experiences to cadaveric training. Several surgeons invited have sent their apologies for being unable to attend due to work commitments, which led authors to believe that past experience with cadaveric dissection was unlikely to be the reason for non-attendance. Nevertheless, this is a possible source of bias that can be addressed in a future randomised trial.

Thiel cadaver training is a good solution to overcome shortfalls of traditionally fixed and fresh frozen cadavers and should be considered as an alternative choice for educationalists designing surgical training sessions.
